# KNCFS: Feature selection for high-dimensional datasets based on improved random multi-subspace learning

**DOI:** 10.1371/journal.pone.0296108

**Published:** 2024-02-23

**Authors:** Cong Guo

**Affiliations:** College of Computer and Information Engineering, Henan University, Kaifeng, China; University of South Australia, AUSTRALIA

## Abstract

Feature selection has long been a focal point of research in various fields.Recent studies have focused on the application of random multi-subspaces methods to extract more information from raw samples.However,this approach inadequately addresses the adverse effects that may arise due to feature collinearity in high-dimensional datasets.To further address the limited ability of traditional algorithms to extract useful information from raw samples while considering the challenge of feature collinearity during the random subspaces learning process, we employ a clustering approach based on correlation measures to group features.Subsequently, we construct subspaces with lower inter-feature correlations.When integrating feature weights obtained from all feature spaces,we introduce a weighting factor to better handle the contributions from different feature spaces.We comprehensively evaluate our proposed algorithm on ten real datasets and four synthetic datasets,comparing it with six other feature selection algorithms.Experimental results demonstrate that our algorithm,denoted as KNCFS,effectively identifies relevant features,exhibiting robust feature selection performance,particularly suited for addressing feature selection challenges in practice.

## 1. Introduction

In disease prediction tasks, the collected DNA microarray datasets are often high-dimensional.Searching for the genes that determine the occurrence of diseases among these genes is challenging,as it constitutes an NP-hard problem with a complexity of O(2^*d*^).Furthermore,in high-dimensional datasets,there exists a significant amount of redundant and noisy features.Blindly learning these features causes the model to learn spurious correlations and reduces the performance of the mode [1, 2].To address this challenge,an effective approach is to reduce data dimensionality through feature selection [3].The objective of feature selection is to retain relevant features while discarding irrelevant ones.Feature selection not only reduces feature dimensionality but also enhances model performance.

Feature selection (FS) methods can be categorized into three primary modes: wrapper mode,filter mode,and embedded mode [4].Wrapper mode typically employs heuristic search to select the most favorable features with respect to evaluation metrics [5–8].Wrapper models typically use swarm intelligent optimisation to generate binary solution vectors,where the selection of a particular feature is denoted by 1 and 0 means that the corresponding feature is not considered in the subset of features.For example,bee colony optimization [6];particle swarm optimization [8];whale optimization [9] etc.However,when dealing with high-dimensional data,these methods often struggle to complete the search within a reasonable time frame [10].To address this issue,some filter-mode methods search for the optimal subset by exploring the intrinsic relationships between samples and features [11–14].For example,literature [13] introduces the correlation coefficient and combines the correlation coefficient and mutual information to measure the relationship between different features for feature selection,and literature [15] uses mutual information and joint mutual information to balance the significance between the two feature correlation terms for weighted correlation-based feature selection.Due to the lack of a specific classifier guiding the feature selection stage,the selected features in such methods may not be optimal [16].On the other hand, embedded mode views the process of learning the optimal subset as an optimization problem.These methods introduce penalties or constraints to FS through the construction of an objective function and regularization terms related to feature weights [17–22].This encourages the model to select the most relevant features.For example,literature [23] embedded the relevance self-representation matrix into unsupervised learning to take into account the complete sample relevance and feature dependencies;literature [24] helped to identify relevant features by embedding indication labels into ridge regression models;and literature [25] proposed a new adaptive LapSVM feature selection method by embedding the acquisition of Laplacian matrices into the SVM training process in order to achieve semi-supervised learning.In comparison to filter mode, embedded mode involves interaction with a classifier and often can select features with the highest information content [26].

The Neighborhood Component Feature Selection (NCFS) [27] is an embedded method in the field of feature selection that has garnered significant attention, primarily due to its excellent performance on high-dimensional datasets.However, NCFS exhibits a notable limitation in that it is confined to acquiring knowledge within the original feature space,leading to a relatively limited information extraction from the raw samples and failing to fully exploit the latent information within the data.In a separate study,a method known as the Random Multi-subspace Approach [28] was proposed.This approach treats the reliefF method as a black box and,through multiple random data set partitions,learns local weights in each subspace to enhance the sample diversity of the reliefF method. However,it is worth noting that the experiments conducted in reference [28] were limited to low-dimensional datasets, with a maximum of 649-dimensional features used. Our further investigation suggests that the direct application of the Random Subspace Approach on high-dimensional datasets offers limited performance enhancement for NCFS.This is because in high-dimensional datasets,some features can be approximately represented as combinations of other features in a linear manner, resulting in a certain degree of feature collinearity.The existence of collinearity can reduce the model’s generalization performance.Furthermore,during the random subspace partitioning of the feature space,a situation may arise where features with collinearity are accidentally assigned to the same subspace.This can lead to model overfitting and consequently decrease the accuracy of feature selection.

In addressing the issue of limited information captured by NCFS from the original samples,we introduce an enhanced approach that simultaneously considers enhancing the diversity of the original samples and mitigating the problem of feature collinearity.In formal terms, we propose a method that utilizes clustering algorithms to construct random subspaces,aiming to alleviate the impact of collinearity. Furthermore,following the completion of feature weight learning within each feature partition,we employ a feature partition weight factor to assess the contribution of each feature partition to the final weight vector, as opposed to a simple averaging approach. Extensive experiments on ten high-dimensional datasets and synthetic datasets validate the effectiveness of our algorithm.The primary contributions of this paper are summarized as follows.

- The proposed method simultaneously addresses the issues of diversity in the random subspace during the feature selection process and feature collinearity.
- A feature partition weight factor is introduced to weight the importance of features learned within each feature partition.
- Multiple sets of experiments on synthetic and real datasets confirm the effectiveness of the proposed method.Notably, the experimental results demonstrate that the consideration of feature collinearity in the random subspace approach enhances the effectiveness of feature selection.

The remainder of the paper is organised as follows,in Section 2 we briefly introduce the NCFS algorithm,2.3 we detail the random multi-subspace method,as well as in 2.4 we briefly introduce the K-means cluster,followed by Section 3 where we present our method, Section 4 shows the experimental results of the new method with the comparative method, and, finally, we draw conclusions in Section 5.

## 2. Preliminaries

In this section,we introduce the notation and definitions of this paper in 2.1.In section 2.2, the original NCFS method is briefly described,and finally 2.3 presents the random multi-subspace method.

### 2.1 Notation and definition

Given a feature matrix **X** = [*x*_1_,*x*_2_,..,*x*_*n*_]^*T*^∈**R**^*n*×*d*^,which is a set of *n* training samples with a dimensionality of *d*,and **y** = [*y*_1_,*y*_2_,..,*y*_*n*_]^*T*^ representing the labels corresponding to the samples,in addition,**X** can be formalised as a feature set *F* = [*f*_1_,*f*_2_,..,*f*_*d*_],where *f*_i_ denotes the column vector consisting of the ith column of features of all samples. Then,according to the definition in [28], the set family *E* is a feature partition of *F* when the following condition holds.

-
∅∉E

-
$\underset{A\in E}{\cup }A=E$

-
∀A,B∈E(A≠B)→A∩B=∅

  when *A* ∈ *E*,*A* is called a random subspace.For example,let *D* = {*f*_1_, *f*_2_, *f*_3_, *f*_4_, *f*_5_} be a set with 5 feature columns,and its subsets are *A* = {*f*_1_, *f*_3_},*B* = {*f*_2_, *f*_4_},and *C* = {*f*_5_},then by definition,{*A*, *B*, *C*} is a feature partition,of which *A*,*B*,*C* is a subspace, respectively.

### 2.2 Neighborhood component feature selection

NCFS is an embedded method for selecting features that utilizes the nearest-neighbour model.It measures the similarity between samples by using feature-weighted distances.Furthermore, for each sample *x*_*i*_,the algorithm measures the probability of correct classification with a probability distribution function.After the probability of all samples being classified correctly being summed up,NCFS then introduces a penalty term to prevent overfitting.

The algorithm initially initializes the feature importance weights **w** as a vector with all elements set to 1.Then,based on **w**,it defines the weighted distance between two samples *x*_*i*_ and *x*_*j*_ as follows:

$$d(x_{i},x_{j})=\sum_{l=1}^{d}{\text{w}}_{l}^{2}|x_{il}-x_{jl}|$$ (1)

where w_*l*_ denotes the weight of the *l*-th feature.In order to learn **w** based on the approximate leave-one-out classification accuracy,the NCFS further gives a definition of the probability that a sample *x*_*i*_ selects *x*_*j*_ as a reference point:

$$p_{ij}=\frac{\kappa (d(x_{i},x_{j}))}{\sum_{k=1,k\neq i}^{n}\kappa (d(x_{i},x_{k}))}$$ (2)

where *κ*(*x*) = *exp*(-*x*/*σ*) is the kernel function and *σ* is the kernel width.According to the above definition,the probability that the query point *x*_*i*_ is correctly classified is:

$$p_{i}=\sum_{j}y_{ij}p_{ij}$$ (3)

where *y*_*ij*_ = 1 if and only if *y*_*i*_ = *y*_*j*_ otherwise *y*_*ij*_ = 0.Finally,NCFS defines the objective function in the following form.

$$\underset{w}{\arg\;\max}F(w)=\sum_{i=1}^{n}p_{i}-\lambda \sum_{l=1}^{d}w_{l}^{2}$$ (4)

where *λ* is the regularisation parameter to be adjusted.For the problem of obtaining the maximum value of the objective function,it is sufficient to make the derivative of the function F(**w**) with respect to **w** equal to 0 to derive the local optimum value of the feature weights **w**,and then use the gradient ascent method to update **w** until *F*(**w**) converges at a point near its maximum value,and output the weighting vector **w** at this point.

### 2.3 Random multi-subspace based learning

The Random Multi-subspace Approach,as introduced in reference [28], involves partitioning the original feature space into *s* different subspaces,with each subspace constructed based on a distinct subset of features.Subsequently,separate feature weight learning is conducted for each of these distinct feature subspaces.By repeatedly performing such partitions,the Random Multi-subspace Approach is capable of extracting additional information from the data,thereby enhancing the model’s robustness and generalization capacity.

In the context of the Random Multi-subspace Approach,each random partition of the original feature space is referred to as a feature partition.Assuming that the Random Multi-subspace Approach conducts *M* random partitions of the original feature space, the ith feature partition can be represented as follows:

$$P^{\left(i\right)}=\left\{P^{\left(i,1\right)},P^{\left(i,2\right)},\ldots ,P^{\left(i,s\right)}\right\}$$ (5)

where *s* represents the number of random subspaces,and *P*^(*i*,*j*)^ signifies the *j*-th subspace within the ith feature partition,*j∈*{1,2, …,*s*}.Here,we assume an equal number of feature subspaces within each feature partition.

For an original feature space comprising d features,to execute a random partition, one can initially generate a random permutation of the *d* features.Subsequently, the first ⌊d/s⌋ features are consecutively assigned to distinct subspaces.The remaining features are sequentially allocated to different subspaces until all features have been partitioned.Evidently,within each feature partition,each feature belongs to a single feature subspace.

For each subspace *P*^(*i*,*j*)^,local feature weights **w**^(*i*,*j*)^ can be computed using feature selection methods such as ReliefF.Then the overall feature weight of the *i*-th feature partition can be obtained by splicing the local weights of its *s* subspaces:

$$w^{\left(i\right)}=\{w^{\left(i,1\right)},w^{\left(i,2\right)},\ldots ,w^{\left(i,s\right)}\}$$ (6)

It is assumed that each feature partition contributes equally to the final feature weight. Then the final feature weights can be obtained by averaging the feature weights of *M* feature partitions:

$$w=\frac{1}{M}\sum_{i=1}^{M}w^{\left(i\right)}$$ (7)

### 2.4 K-means cluster

We employ the k-means algorithm for feature clustering,which is one of the most well-known and widely used clustering methods [29]. It partitions a set of samples into *k* clusters (the value of *k* needs to be predetermined).

Let *A* = {*a*_1_, . .. , *a*_*k*_} represent the k cluster centers.Consider **z** = [*z*_*ic*_ ]^*d*×*c*^,where z_*ic*_ is a binary variable taking values 0 or 1,indicating whether feature *f*_*i*_ belongs to the c-th cluster,where *c* = {1, …, *k*}.The objective function of *k*-means is given by:

$$\min\;J(A,z)=\sum_{i=1}^{d}\sum_{c=1}^{k}z_{ic}D^{2}(f_{i}-a_{c})$$ (8)

where *D*^2^(*f*_*i*_-*a*_c_) denotes the Euclidean distance between feature *f* and the *c*-th cluster centre *a*_*c*_.The Euclidean distance is a commonly used similarity measure.The k-means algorithm iteratively minimizes the objective function *J*(*A*, **z**) and updates the cluster centers *A* and the membership matrix **z** as follows:

$$a_{c}=\frac{\sum_{i=1}^{d}z_{ic}f_{i}}{\sum_{i=1}^{d}z_{ic}}$$ (9)

$$z_{ic}=\left\{\begin{array}{c} 1\;\text{if}\;1-|\text{pea}(f_{i}-a_{c})|=\underset{1\leq c\leq k}{\min}D^{2}(f_{i}-a_{c})\\ 0,\text{otherwise}. \end{array}\right.$$ (10)

The algorithmic steps of K-means are as follows:Initially, *k* features are randomly chosen as the centers of the *k* clusters.Then,(1) the membership degree of each feature to each cluster center is computed following Eq (10). For a feature *f*_*i*_, it is assigned to cluster *a*_*c*_ if *a*_*c*_ is its nearest cluster center.Once all features are assigned to their respective clusters,(2) the positions of each cluster center are updated following Eq (9). Steps 1 and 2 iterate mutually until the stopping criteria are met.

## 3. The proposed method

Previous random multi-subspace weight learning methods did not take into account that in high-dimensional datasets,some highly collinear features might be accidentally allocated to the same subspace.This collinearity is prevalent and can lead to local overfitting of the algorithm,thereby reducing the accuracy of feature selection. Hence,to address the issue of feature collinearity within random subspaces while ensuring diversity within the original sample space,we propose a novel approach.

**Fig 1:**
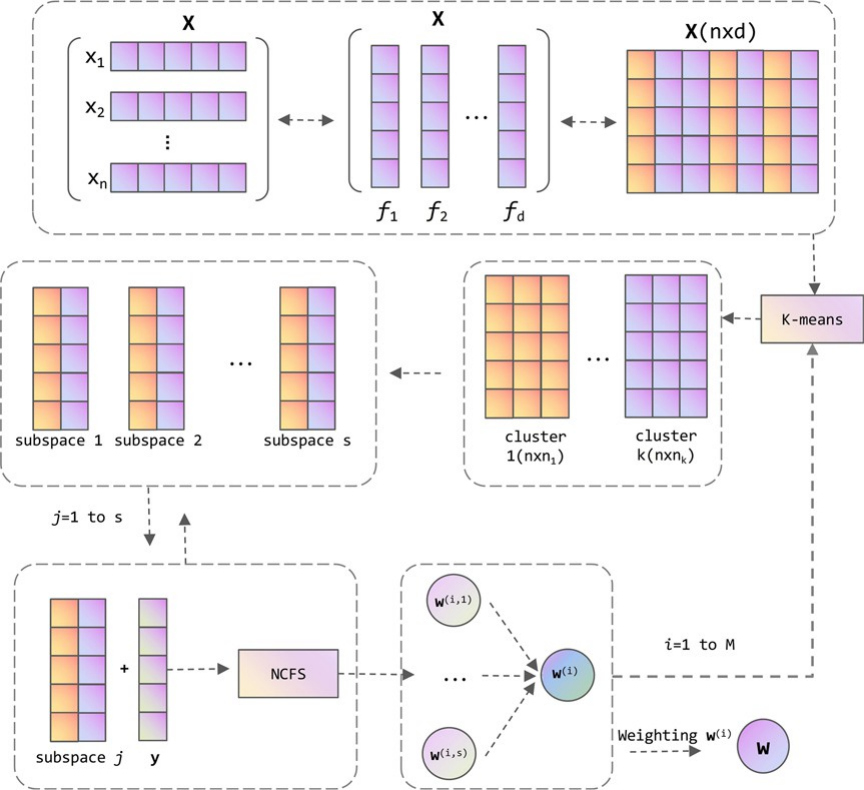
Overall framework diagram of the algorithm.

Our algorithm requires performing *M* iterations.Initially,each feature is treated as equally important,with the initial weight vector **w** set as a vector of all ones. In the *i*-th iteration of the algorithm,we commence by partitioning the original feature set into *k* clusters based on a correlation measure using K-means clustering.Subsequently, we randomly select features from each feature cluster to construct *s* equally sized random subspaces.Within each subspace,we employ NCFS to learn its local feature weights. These local feature weights from each subspace are then integrated to form a complete *d*-dimensional feature vector,denoted as **w**^(*i*)^.When integrating the feature weights **w**^(*i*)^ learned during each iteration into the overall feature weight vector **w**,we apply an importance factor to weight them,as opposed to the previous approach of taking a simple average.The general framework of the algorithm is illustrated in Fig 1. Section 3.1 presents the method for constructing random subspaces using K-means clustering,while Section 3.2 introduces the proposed weighting factor.

### 3.1 Use K-means to generate subpsaces

To address the issue of feature collinearity within random subspaces,we cluster features based on their inter-correlations.The purpose of this step is to group features with a certain level of correlation into the same cluster.To achieve this,we employ the correlation coefficient as a measure in the K-means objective function instead of the Euclidean distance.Consequently, the formulas for Eqs (8) and (10) should be defined as follows:

$$\begin{array}{l} \min\;J(A,z)=\sum_{i=1}^{d}\sum_{c=1}^{k}z_{ic}\left(\text{1-}|\text{pea}(f_{i}-a_{c})|\right)\\ z_{ic}=\left\{\begin{array}{c} 1\;\text{if}\;1-|\text{pea}(f_{i}-a_{c})|=\underset{1\leq c\leq k}{\min}\left(\text{1-}|\text{pea}(f_{i}-a_{c})|\right)\\ 0,\text{otherwise}. \end{array}\right. \end{array}$$ (11)

where,pea(*f*_*i*_-*a*_*c*_) represents the Pearson coefficient between feature *f*_*i*_ and the *c*-th cluster center, *a*_*c*_.The Pearson coefficient is a commonly used measure of the degree of correlation between variables and its values range between -1 and 1.A Pearson coefficient closer to 0 indicates a smaller degree of correlation between variables, while a coefficient closer to 1 (or -1) suggests a larger degree of correlation.

Once the feature set is divided into *k* clusters (*K*_1_, …, *K*_*k*_),for any feature cluster *K*_*c*_, it comprises different features, denoted as:

$$K_{c}=\{f_{\left(c,1\right)},\ldots ,f_{\left(c,n_{c}\right)}\}$$ (12)

where *f*_(*c*,*j*)_ represents the *j-*th feature in the *c*-th feature cluster,and *n*_*c*_ is the number of features in the *c*-th feature cluster.To construct feature subspaces with low collinearity among their constituents,we generate a random permutation of length *n*_*c*_ for each feature cluster *K*_*c*_.Subsequently,we sequentially assign $\left\lfloor n_{c}/s\right\rfloor$ contiguous features to each subspace.The remaining features are assigned to different subspaces in order until all features have been partitioned. Therefore,the feature cluster *K*_*c*_ can be represented as:

$$K_{c}=\{su^{\left(c,1\right)},\ldots ,su^{\left(c,s\right)}\}$$ (13)

where *su*^(*c*,*j*)^denotes the feature assigned by *K*_*c*_ to the *j-*th subspace,and any intersection between *su*^(*c*,*j*)^ is empty.Thus,for each subspace *P*^(*i*,*j*)^,it can be represented as:

$$P^{\left(i,j\right)}=\{su^{\left(1,j\right)},\ldots ,su^{\left(k,j\right)}\}$$ (14)

each feature cluster is evenly divided into *s* segments,which are then separately incorporated into each subspace.Each subspace,denoted as *P*^(*i*,*j*)^,constitutes the *i*-th feature partition generated by the algorithm.Subsequently,we employ NCFS to learn local feature weights within each subspace,where **w**^(*i*,*j*)^ represents the feature weights learned in the *j*-th subspace of the *i*-th feature partition (i.e., the *i*-th iteration of the algorithm).Upon computing the feature weights **w**^(*i*,*j*)^ for all subspaces,they are consolidated into a complete *d*-dimensional feature weight vector,denoted as **w**^(*i*)^.

### 3.2 Weighting w^(i)^

Previously,every feature partition’s contribution was assigned equal weight in prior methods for random subspace,resulting in an average of weights across all partitions in the final feature weights outcome.Our argument is that contributions from each feature partition may not all have the same weight.Therefore, we introduce an appraisal factor,*α*,for the feature weight vector, **w**^(*i*)^,to provide a weighted assessment for every **w**^(*i*)^.Before calculating *α*,it is crucial to acquire a correlation matrix **R** linked to the feature matrix **X**.To compute the (*i*, *j*)-th element of **R**,use the subsequent equation:

$$R_{ij}=\text{pea}(f_{i},f_{j})$$ (15)

where *f*_*i*_ and *f*_*j*_ represent the *i*-th and *j*-th feature columns of **X**,‘pea’ denotes the Pearson coefficient.According to the above definition,**R** is a *d*×*d* symmetric matrix. Subsequently,we perform Cholesky decomposition on **R** and multiply it by a random sample θ drawn from a Gaussian distribution,resulting in **v**:

v=Cholesky(R)×θ (16)

The form of **v** conforms to **w**,a chance variable that maintains a particular correlation structure.Afterward,we introduce **v** into a cumulative multivariate distribution function,resulting in a cumulative multivariate probability **u** that is distributed across [0–1]:

$$u=\frac{1}{2}(1+\frac{2}{\sqrt{\pi }}\int_{0}^{v}\exp(-t^{2})dt)$$ (17)

Define **π**^(*i*)^as the normalised **w**^(*i*)^ and **X**^(*i*)^ as an empty set.Formally,if (**π**^(*i*)^)_*k*_ is greater than *u*_*k*_, the feature *f*_*k*_ is added to **X**^(*i*)^.Finally,the weighting factor *α* is defined as:

$$\alpha (w)=evaluate(classifier,X^{\left(i\right)},y)$$ (18)

In this case,the ’evaluate’ function calculates the classification results on the feature matrix **X**^(*i*)^,and the classification accuracy (ACC) can be selected as the evaluation criterion,with Classifier representing the selected classifier.We define ’classifier’ as the KNN classifier(*k* = 3).Therefore,at the end of the *i*-th iteration of the algorithm,we use the following formula to add **w**^(*i*)^ to the total weight vector **w**:

$$w=w+\alpha (w^{\left(i\right)})\times w^{\left(i\right)}$$ (19)

where **w**^(*i*)^ denotes the feature weight vector obtained by the algorithm from the *i*-th iteration.The detailed steps of the algorithm are described in Algorithm 1.

**Algorithm 1**:K-means Neighbourhood Component Feature Selection(KNCFS)
**Input**:Feature matrix:**X**(*x*_1_,*x*_2_, …,*x*_*n*_)^*T*^ = (*f*_1_,*f*_2_, …,*f*_*d*_),labels corresponding to the samples:***y***(*y*_1_,*y*_2_, …,*y*_*n*_),*M*:number of feature partitions,*s*:number of subspaces for each feature partirions,*k*:number of feature clusters
**Output:**feature importance vector w
1 Initialisation:**w** = (1,1, …,1)^*d*^,*F* = {*f*_1_, …,*f*_*d*_}.
2 ***for*** *i* = 1 to *M* ***do***
3 set *P*^(*i*,1)^, …,*P*^(*i*,s)^ as sempty sets.
4 *K*_1_, …,*K*_*k*_ = K-means(*F*,*k*).
5 ***for*** *c* = 1 to *k* ***do***
6  ***for*** *j* = 1 to *s* ***do***
7  randomly select $\left\lfloor len(K_{c})/s\right\rfloor$ features from *K*_*c*_,add them into *P*^(*i*,*j*)^,and remove the selected features in *K*_*c*_.
8  ***if*** *K*_*c*_ is not empty ***then***
9   ***for*** *q* = 1 to len(*K*_*c*_) ***do***
10    Randomly select a feature from *K*_*c*_ to be added to any subspace *P*^(*i*,*s*)^, and subsequently remove this feature from *K*_*c*_.
11 ***for*** *j* = 1 to *s* ***do***
12  use NCFS to compute feature importance **w**^(*i*,*j*)^ on *P*^(*i*,*j*)^.
13  **w**^(*i*,1)^, …,**w**^(*i*,*s*)^ are fromed into a complete weighting vector **w**^(*i*)^ based on the indexing of the features.
14 $\Pi^{\left(i\right)}=\frac{w^{\left(i\right)}-min(w^{\left(i\right)})}{max(w^{\left(i\right)})-min(w^{\left(i\right)})}.$
15 Compute **R**,**v**,**u** according to Eqs (15–17) and set **X**^(*i*)^ as an empty set.
16 **if** (**π**^(i)^)_k_ >u_k_ **then**
17  add *f*_*k*_ into **X**^(*i*)^.
18 caculate *α* according to Eq (18).
19 **w** = **w**+*α*(**w**^(*i*)^)×**w**^(*i*)^.
20 return **w**

## 4. Experiments

In this section,we conducted multiple sets of experiments to evaluate the performance of the proposed algorithm.Firstly,we explore the convergence of the proposed K-means algorithm based on Pearson coefficients.Subsequently,we compared this method with other approaches on both synthetic datasets and real-world datasets,and the experimental results confirmed the effectiveness of this method.Finally,we investigated the sensitivity of the algorithm to its parameters to determine the optimal parameter configuration.

### 4.1 Datasets

Ten real-world datasets were utilised as the primary experimental benchmarks to assess the performance of the proposed method.These datasets were obtained from diverse domains such as facial images (pixraw10P, warpAR10P),biomedical data (lung_discrete,tumors_C,GLIOMA,TOX_171,leukemia,ALLAML),and other areas (SCADI,arcene). Table 1 offers comprehensive information on these datasets.In addition,synthetic datasets were produced as benchmarks utilizing four small-scale datasets from the UCI database [30]. Further details about these synthetic datasets will be explained in Section 4.5.2. Subsequently,all datasets were normalized to conform to a standard distribution.

**Table 1:** Dataset.

Dataset	samples	features	classes	sourse
SCADI	62	206	2	UCI
lung_discrete	73	325	7	https://jundongl.github.io/scikit-feature/datasets.html
warpAR10P	130	2400	10	https://jundongl.github.io/scikit-feature/datasets.html
tumors_C	60	7129	2	https://www.openml.org/
GLIOMA	50	4434	4	https://jundongl.github.io/scikit-feature/datasets.html
TOX_171	171	5748	4	[16]
leukemia	72	7070	2	[31]
ALLAML	72	7129	2	https://jundongl.github.io/scikit-feature/datasets.html
pixraw10P	100	10000	10	[31]
arcene	200	10000	2	UCI

### 4.2 Compared method

We compared the proposed KNCFS method against six different approaches. Specifically,we used chi-square as a baseline and we compared KNCFS with two widely used feature selection (FS) methods [32–34], Fisher-score and ReliefF. Additionally,we considered RBEFF,recognized as one of the most advanced FS methods,and NCFS,renowned for its excellent performance on high-dimensional datasets.Given the various improvements we made to NCFS,to evaluate the effectiveness of the improvements in this paper,we also used RB-NCFS as a comparative method.Below,we provide a brief overview of all the comparative methods:

- chi-square [35]: A statistical method used to select categorical variables significantly associated with the target variable.
- fisher-score [36]: Measures the importance of features for classification tasks by comparing the variance between different classes and within classes.
- ReliefF [37]: A feature selection method based on a nearest neighbor model,calculated using reliefF scores to assess feature importance,Number of nearest neighbours *k* = 5.
- RBEFF [28]: A method based on random subspaces that uses reliefF to learn local feature weights in subspaces,number of feature partitions *M* = 10,number of subspaces *s* = 10,number of nearest neighbours in reliefF *k* = 5.
- NCFS [27]: A feature selection method based on fast neighborhood model analysis,maximizing leave-one-out classification accuracy to obtain feature weights,the kernel width *σ* = 1 and the regularisation parameter *λ* = 1.
- RB-NCFS:A method based on random subspaces that uses NCFS to learn local feature weights in subspaces,number of feature partitions *M* = 10,number of subspaces *s* = 10,the kernel width for NCFS *σ* = 1 and the regularisation parameter *λ* = 1.

### 4.3 Compared metric

In our experiments,to validate the effectiveness of the method,we employed four classifiers to calculate classification performance:Support Vector Machine (SVM), Naive Bayes (NB),Decision Tree (DT),and K-Nearest Neighbors (KNN).Additionally, we utilized standard evaluation metrics,accuracy (ACC),and *F*_1_-score,to assess the performance of different feature selection methods.ACC and *F*_1_-score range between 0 and 1,where higher values indicate better performance.

1. ACC:

$$ACC(X,y)=\frac{1}{n}\sum_{i=1}^{n}\text{I}(y_{i}=c(x_{i}))$$ (20)

Where I(*y*_i_ = *c*(*x*_i_)) = 1 if and only if *y*_i_ = *c*(*x*_i_),*y*_*i*_ is the true label of *x*_*i*_,*c*(*x*_*i*_) is the predicted label of sample *x*_*i*_ by the classifier.

2. *F*_*1*_-score:

In binary classification problems,samples are categorized into four scenarios based on the actual labels and predicted labels:True Positives (TP),False Positives (FP),True Negatives (TN),and False Negatives (FN).Precision is the proportion of samples predicted as "positive" that are actually "positive" among all samples predicted as "positive," while recall is the proportion of samples actually labeled as "positive" that were correctly predicted as "positive" by the model.The definitions of these two metrics are as follows (Eqs (21) and (22)):

$$precision=\frac{TP}{TP+FP}$$ (21)

$$recall=\frac{TP}{TP+FN}$$ (22)

Often,we would like to take care of both Precision and Recall,therefore,*F*_1_-Score is another commonly used metric,which is the reconciled average of Precision and Recall,and can be used to comprehensively evaluate the performance of the model. It is defined in Eq (23):

$$F_{1}=2\cdot \frac{precision\cdot recall}{precision+recall}$$ (23)

The *F*_1_-score for binary classification can be extended to multiclassification problems,where one of the classes is considered as a positive class and the others as negative classes,and then the *F*_1_-Score is calculated according to Eq (23).

### 4.4 Parameter settings

For KNCFS,there are three parameters to consider:the number of feature subspaces *M*,the number of subspaces *s*,the number of clusters for feature clustering *k*. In prior research,the values for *σ* and *λ* in NCFS were recommended to be {1, 1},and for *M*,*s*,and *k*,we will explore their optimal settings within the range of {5, 10, 15, 20, 25}.

Regarding the parameters for classifiers,we chose the RBF kernel for the support vector machine,set the maximum tree depth to 5 for the decision tree,and selected 3 nearest neighbors for K-nearest neighbors (KNN).The parameter configurations are summarized in Table 2.

**Table 2:** Parameter settings.

Parameter	Settings
*t* The number of runs	10
*K* for KNN classifier	3
Max_dept for DT classifier	5
Kernel function of SVM	RBF
*σ* for NCFS	1
*λ* for NCFS	1
*M* the number of feature partitions	5–20
*s* the number of subspaces	5–20
*k* the number of clusters	5–20

### 4.5 Results

#### 4.5.1 Convergence results

**Fig 2:**
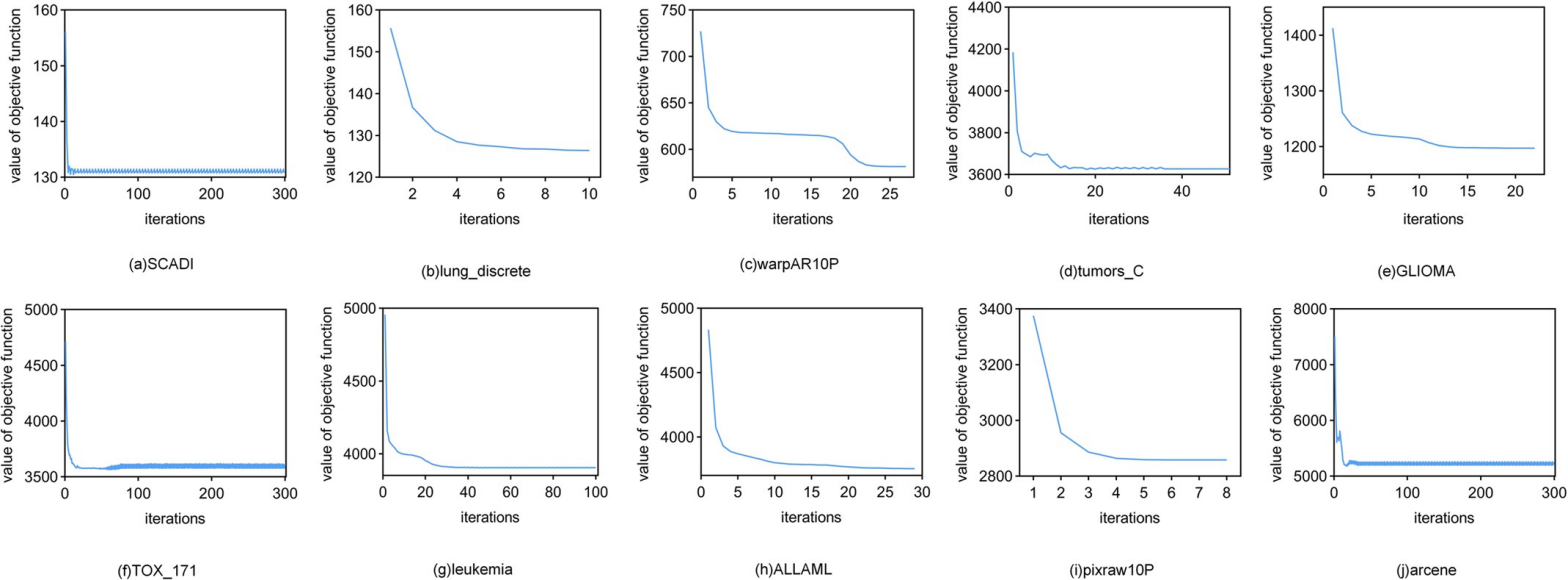
Convergence curves of K-means.

In this paper,K-means obtains the optimal result under the condition that:at the kth iteration,the objective function *J*_*k*_(*A*,**z**)-*J*_(*k-*1)_(*A*,**z**)<*η* or *k* > max_iter where we set *η* = 0.02 and max_iter = 300. Fig 2 shows the convergence of the K-means method we use on ten datasets,and we find that that the algorithm converges quickly on most datasets,while on the SCADI,TOX_171,and arcene datasets,the algorithm’s objective function value oscillates within an interval,and returns results only when the maximum number of iterations is reached.

#### 4.5.2 Classification result

**Fig 3:**
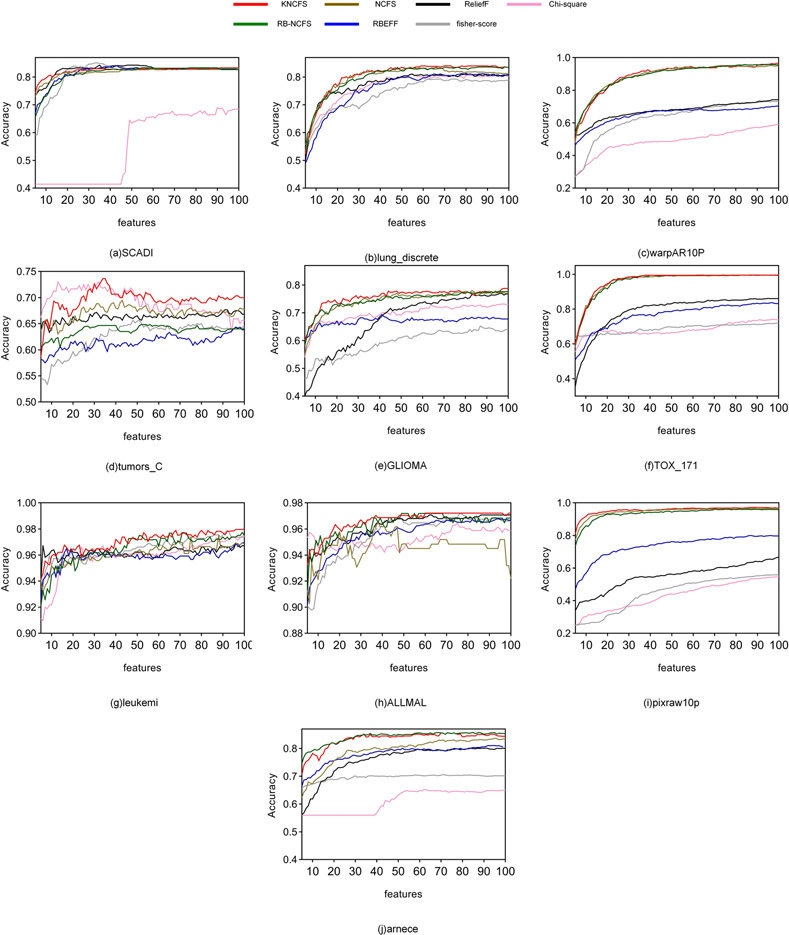
Classification results with SVM on 10 datasets. Selected 5–100 features.

**Fig 4:**
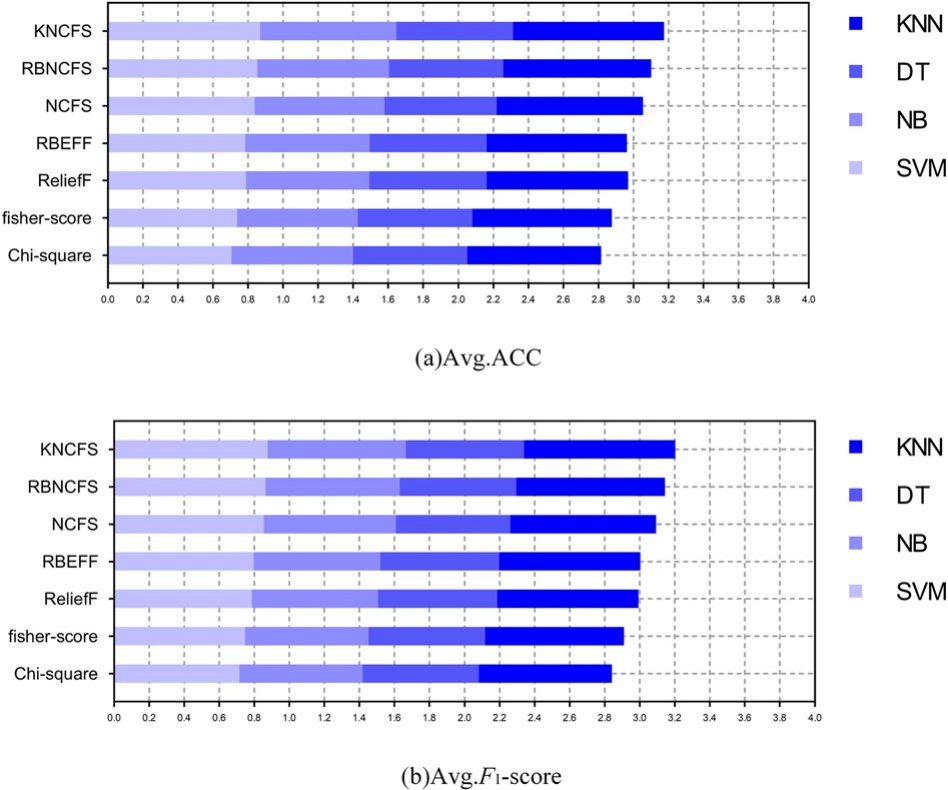
Average classification results with 4 classifiers on 10 datasets.

The average classification accuracy of the seven feature selection methods is presented in Tables 3 and 4,while the *F*_1_-score results are shown in Tables 5 and 6. The last row in the tables demonstrates the number of times each method achieved the best results (win/tie).The average results acheaved by SVM with defferent features are shown in Fig 3.The average results accumulated by the four classifiers are shown in Fig 4.

**Table 3:** ACC of 10-fold cross validation on 10 datasets (Mean±std). Selected 50 features.

Dataset		Chi-square	Fisher-score	ReliefF	RBEFF	NCFS	RB-NCFS	KNCFS
SCADI	SVM	0.657±0.01	0.828±0.01	0.828±0.01	**0.83±0.01**	0.825±0.01	0.828±0.01	0.829±0.01
	NB	0.638±0.02	0.775±0.01	0.795±0.02	**0.795±0.02**	0.784±0.02	0.792±0.01	0.79±0.02
	DT	0.69±0.02	0.78±0.02	0.781±0.03	0.783±0.01	0.768±0.02	0.785±0.03	**0.789±0.02**
	KNN	0.67±0.01	0.804±0.01	0.81±0.01	**0.814**±0.01	0.808±0.02	0.797±0.01	0.804±0.01.
lung_discrete	SVM	0.769±0.05	0.763±0.03	0.79±0.02	0.772±0.04	0.803±0.02	0.815±0.03	**0.823±0.02**
	NB	0.574±0.04	0.584±0.03	0.626±0.03	0.628±0.03	0.595±0.04	0.608±0.02	**0.663±0.03**
	DT	0.524±0.05	0.592±0.04	0.52±0.04	0.529±0.04	0.552±0.06	0.562±0.04	**0.565±0.04**
	KNN	0.802±0.04	0.817±0.03	0.814±0.03	0.791±0.04	0.826±0.02	0.824±0.02	**0.845±0.02**
warpAR10P	SVM	0.487±0.03	0.706±0.02	0.681±0.03	0.677±0.02	**0.919±0.01**	0.87±0.02	0.906±0.04
	NB	0.637±0.02	0.686±0.01	0.638±0.02	0.64±0.02	0.807±0.02	0.775±0.02	**0.809±0.02**
	DT	0.526±0.04	0.481±0.04	0.626±0.04	0.652±0.04	0.472±0.03	0.552±0.06	**0.558±0.05**
	KNN	0.621±0.03	0.757±0.02	0.767±0.02	0.746±0.03	0.865±0.02	0.833±0.02	**0.893±0.02**
tumors_C	SVM	**0.72±0.02**	0.648±0.03	0.671±0.03	0.628±0.03	0.64±0.01	0.612±0.04	0.65±0.03
	NB	**0.691±0.02**	0.6±0.03	0.625±0.02	0.608±0.03	0.455±0.01	0.506±0.05	0.641±0.04
	DT	**0.631±0.05**	0.615±0.06	0.553±0.05	0.581±0.04	0.56±0.05	0.562±0.04	0.577±0.05
	KNN	0.611±0.03	0.61±0.03	0.603±0.05	0.613±0.04	0.492±0.03	0.577±0.05	**0.618±0.06**
GLIOMA	SVM	0.7±0.02	0.614±0.05	0.674±0.03	0.692±0.02	0.77±0.03	0.776±0.02	**0.792±0.01**
	NB	0.576±0.05	0.624±0.03	0.618±0.05	0.646±0.03	0.702±0.04	0.686±0.04	**0.718±0.03**
	DT	0.632±0.05	0.52±0.06	0.654±0.05	0.598±0.06	**0.674**±0.05	0.618±0.06	0.662±0.07
	KNN	0.694±0.07	0.67±0.05	0.69±0.04	0.694±0.02	0.792±0.03	**0.816**±0.03	0.8±0.02
TOX_171	SVM	0.659±0.04	0.698±0.02	0.84±0.02	0.78±0.03	0.962±0.01	0.993±0.01	**0.995±0.01**
	NB	0.611±0.03	0.607±0.01	0.686±0.03	0.702±0.02	0.745±0.02	0.761±0.03	**0.79±0.02**
	DT	0.55±0.03	0.544±0.01	0.596±0.03	0.553±0.03	0.605±0.02	0.613±0.02	**0.628±0.02**
	KNN	0.739±0.02	0.743±0.01	0.887±0.03	0.803±0.03	0.944±0.01	0.964±0.01	**0.973±0.01**
leukemia	SVM	0.958±0.01	0.96±0.01	0.953±0.01	0.96±0.01	0.968±0.01	0.969±0.01	**0.975±0.01**
	NB	0.947±0.01	0.938±0.01	0.931±0.01	0.934±0.01	0.942±0.01	0.946±0.01	**0.95±0.01**
	DT	0.931±0.01	0.935±0.01	0.941±0.01	0.937±0.01	0.931±0.01	0.935±0.01	**0.944±0.01**
	KNN	0.965±0.01	0.95±0.01	0.948±0.01	0.947±0.01	0.962±0.01	0.966±0.01	**0.972±0.01**
ALLAML	SVM	0.945±0.01	0.964±0.01	0.968±0.01	0.968±0.01	0.94±0.01	**0.97±0.01**	0.967±0.01
	NB	0.957±0.01	0.956±0.01	0.946±0.02	0.956±0.01	0.955±0.02	0.967±0.01	**0.978**±0.01
	DT	0.862±0.03	0.854±0.02	0.865±0.04	0.853±0.03	0.851±0.03	0.862±0.03	**0.868**±0.03
	KNN	0.909±0.01	0.947±0.01	0.923±0.01	0.931±0.01	0.933±0.01	0.946±0.01	**0.96**±0.01
pixraw10P	SVM	0.447±0.06	0.51±0.06	0.771±0.05	0.768±0.04	0.95±0.01	0.953±0.01	**0.955±0.02**
	NB	**0.869±0.03**	0.618±0.03	0.681±0.02	0.686±0.04	0.841±0.01	0.857±0.03	0.74±0.03
	DT	0.589±0.05	0.53±0.07	**0.59**±0.08	0.549±0.04	0.55±0.04	0.477±0.05	0.479±0.03
	KNN	0.87±0.02	0.939±0.01	0.844±0.03	0.862±0.03	0.927±0.02	0.939±0.01	**0.961±0.01**
arcene	SVM	0.631±0.02	0.708±0.01	0.796±0.02	0.798±0.02	0.81±0.01	0.825±0.01	**0.837**±0.01
	NB	0.622±0.02	0.681±0.01	0.655±0.03	0.686±0.02	0.745±0.01	0.732±0.01	**0.753**±0.01
	DT	0.632±0.02	0.675±0.02	0.727±0.03	**0.744±0.02**	0.696±0.02	0.728±0.02	0.7±0.03
	KNN	0.616±0.02	0.707±0.02	0.808±0.02	0.809±0.01	0.816±0.02	0.84±0.02	**0.843**±0.01
W/T/L		4/0	0/0	1/0	4/0	2/0	2/0	27/0

**Table 4:** ACC of 10-fold cross validation on 10 datasets (Mean±std). Selected 100 features.

Dataset		Chi-square	Fisher-score	ReliefF	RBEFF	NCFS	RB-NCFS	KNCFS
SCADI	SVM	0.655±0.01	0.83±0.01	0.83±0.01	0.828±0.01	0.825±0.01	0.83±0.01	**0.833±0.01**
	NB	0.632±0.02	0.801±0.01	0.804±0.02	0.79±0.01	0.775±0.01	0.798±0.01	**0.807±0.01**
	DT	0.71±0.02	**0.78±0.02**	0.774±0.04	**0.78±0.02**	0.758±0.04	0.773±0.03	0.762±0.03
	KNN	0.688±0.02	0.805±0.01	0.802±0.01	**0.808±0.01**	0.8±0.01	0.801±0.01	**0.808±0.01**
lung_discrete	SVM	0.81±0.03	0.789±0.02	0.78±0.01	0.806±0.02	0.816±0.02	0.819±0.02	**0.823±0.02**
	NB	0.585±0.02	0.572±0.04	0.555±0.04	0.595±0.03	0.589±0.03	0.586±0.02	**0.639±0.03**
	DT	0.539±0.04	0.543±0.04	0.542±0.04	0.511±0.05	0.562±0.05	0.564±0.04	**0.585±0.06**
	KNN	0.835±0.03	0.812±0.03	0.816±0.03	**0.859±0.03**	0.832±0.01	0.845±0.03	0.853±0.02
warpAR10P	SVM	0.62±0.03	0.726±0.02	0.762±0.02	0.694±0.02	0.943±0.02	0.95±0.01	**0.965±0.01**
	NB	0.711±0.02	0.713±0.01	0.718±0.02	0.653±0.02	0.813±0.02	0.815±0.04	**0.823±0.04**
	DT	**0.7±0.04**	0.676±0.03	0.666±0.02	0.659±0.02	0.469±0.02	0.594±0.03	0.556±0.05
	KNN	0.731±0.03	0.796±0.01	0.799±0.03	0.768±0.02	0.876±0.03	0.901±0.02	**0.937±0.02**
tumors_C	SVM	0.65±0.03	0.653±0.03	0.66±0.03	0.646±0.02	0.635±0.02	0.575±0.04	**0.662±0.05**
	NB	0.641±0.03	0.626±0.03	0.613±0.04	0.603±0.03	0.541±0.04	0.516±0.07	**0.654±0.05**
	DT	**0.615±0.08**	0.596±0.06	0.573±0.05	0.603±0.04	0.501±0.08	0.531±0.06	0.564±0.08
	KNN	0.576±0.03	**0.651±0.05**	0.616±0.03	0.598±0.04	0.586±0.02	0.554±0.05	0.597±0.05
GLIOMA	SVM	0.726±0.04	0.62±0.04	0.644±0.05	0.688±0.02	**0.782±0.03**	0.775±0.03	**0.782±0.02**
	NB	0.514±0.04	0.656±0.05	0.651±0.03	0.636±0.04	0.672±0.04	**0.685±0.03**	**0.685±0.05**
	DT	0.614±0.06	0.634±0.06	0.646±0.06	0.586±0.08	0.64±0.05	0.622±0.08	**0.667±0.01**
	KNN	0.746±0.03	0.684±0.04	0.695±0.03	0.702±0.05	0.796±0.03	0.79±0.02	**0.805±0.02**
TOX_171	SVM	0.74±0.03	0.725±0.04	0.863±0.02	0.832±0.02	0.993±0.01	0.99±0.01	**0.996±0.01**
	NB	0.633±0.02	0.619±0.02	0.812±0.03	0.743±0.02	0.809±0.04	0.802±0.02	**0.828±0.03**
	DT	0.567±0.04	0.564±0.03	0.551±0.05	0.56±0.03	0.611±0.03	**0.649±0.04**	0.621±0.03
	KNN	0.833±0.02	0.767±0.03	0.904±0.02	0.87±0.03	**0.984±0.02**	0.953±0.01	0.983±0.01
leukemia	SVM	0.973±0.01	0.976±0.01	0.977±0.01	0.964±0.01	0.973±0.01	0.978±0.01	**0.98±0.01**
	NB	0.921±0.01	0.931±0.01	0.933±0.01	0.93±0.02	0.931±0.01	**0.934±0.02**	**0.934±0.02**
	DT	0.938±0.02	0.936±0.02	0.936±0.01	0.931±0.02	0.939±0.01	0.939±0.02	**0.941±0.01**
	KNN	0.965±0.01	0.965±0.02	0.962±0.01	0.953±0.01	0.974±0.01	0.972±0.01	**0.98±0.01**
ALLAML	SVM	0.964±0.01	0.966±0.01	0.96±0.01	**0.97±0.01**	0.77±0.02	0.966±0.01	**0.97±0.01**
	NB	0.852±0.01	0.958±0.01	0.958±0.01	0.955±0.01	0.771±0.02	0.964±0.01	**0.981±0.01**
	DT	0.848±0.03	0.832±0.03	0.838±0.02	**0.853±0.04**	0.726±0.04	0.834±0.02	0.841±0.03
	KNN	0.917±0.01	0.94±0.01	0.941±0.01	0.928±0.02	0.762±0.02	0.94±0.01	**0.956**±0.01
pixraw10P	SVM	0.55±0.03	0.586±0.04	0.583±0.04	0.802±0.05	0.962±0.01	**0.97±0.07**	0.96±0.01
	NB	0.8±0.03	0.482±0.03	0.465±0.04	0.576±0.04	0.81±0.04	0.801±0.04	**0.811±0.04**
	DT	**0.536±0.05**	0.532±0.04	0.521±0.03	0.531±0.03	0.478±0.08	0.375±0.05	0.456±0.07
	KNN	0.905±0.01	0.948±0.01	0.942±0.01	0.898±0.04	0.941±0.01	**0.951±0.01**	0.94±0.01
arcene	SVM	0.649±0.01	0.689±0.02	0.709±0.02	0.818±0.02	0.816±0.02	**0.843±0.02**	0.835±0.01
	NB	0.636±0.01	0.67±0.01	0.677±0.01	0.718±0.02	0.769±0.01	0.757±0.01	**0.78±0.02**
	DT	0.64±0.02	0.68±0.03	0.7±0.03	0.739±0.02	0.72±0.02	**0.74±0.03**	0.708±0.04
	KNN	0.65±0.02	0.701±0.01	0.715±0.02	0.837±0.02	0.838±0.01	**0.859±0.02**	0.843±0.03
W/T/L		3/0	1/0	0/0	4/0	1/1	8/1	21/5

**Table 5:** *F*_1_-score of 10-fold cross validation on 10 datasets (Mean±std). Selected 50 features.

Dataset		Chi-square	Fisher-score	ReliefF	RBEFF	NCFS	RB-NCFS	KNCFS
SCADI	SVM	0.645±0.01	0.799±0.01	0.805±0.01	0.805±0.01	0.802±0.01	0.807±0.01	**0.808±0.01**
	NB	0.638±0.02	0.756±0.02	0.777±0.02	**0.78±0.02**	0.764±0.02	0.771±0.01	0.768±0.02
	DT	0.688±0.01	0.77±0.02	0.77±0.03	0.775±0.02	0.763±0.03	0.761±0.02	**0.778±0.03**
	KNN	0.701±0.02	0.788±0.02	0.797±0.01	0.8±0.01	0.793±0.01	0.787±0.01	**0.806±0.01**
lung_discrete	SVM	0.742±0.04	0.73±0.03	0.766±0.03	0.749±0.06	0.793±0.02	0.796±0.02	**0.826±0.02**
	NB	0.517±0.05	0.528±0.03	0.576±0.04	0.575±0.04	0.546±0.04	0.57±0.06	**0.629±0.04**
	DT	0.519±0.05	0.59±0.05	0.503±0.04	0.528±0.04	0.551±0.05	0.567±0.04	**0.573±0.05**
	KNN	0.803±0.04	0.8±0.03	0.799±0.03	0.773±0.05	0.824±0.03	0.82±0.02	**0.839±0.02**
warpAR10P	SVM	0.464±0.02	0.706±0.02	0.66±0.03	0.663±0.02	**0.92±0.01**	0.863±0.02	0.905±0.02
	NB	0.631±0.03	0.69±0.02	0.616±0.03	0.615±0.02	**0.814±0.02**	0.763±0.03	0.802±0.02
	DT	0.498±0.05	0.438±0.04	0.59±0.04	0.63±0.04	0.444±0.04	0.507±0.05	0.52±0.03
	KNN	0.612±0.04	0.754±0.02	0.759±0.02	0.743±0.03	0.865±0.01	0.831±0.01	**0.866±0.03**
tumors_C	SVM	**0.713±0.02**	0.634±0.03	0.638±0.02	0.587±0.03	0.528±0.04	0.526±0.04	0.607±0.04
	NB	0.628±0.03	0.612±0.02	0.621±0.03	0.607±0.03	0.437±0.02	0.475±0.06	**0.632±0.04**
	DT	**0.631**±0.06	0.618±0.05	0.545±0.06	0.585±0.04	0.483±0.04	0.546±0.04	0.571±0.05
	KNN	**0.616**±0.03	0.604±0.03	0.57±0.05	0.557±0.03	0.479±0.03	0.514±0.04	0.595±0.04
GLIOMA	SVM	0.679±0.04	0.694±0.02	0.647±0.03	0.684±0.02	0.769±0.04	0.771±0.02	**0.782±0.01**
	NB	0.573±0.05	0.594±0.02	0.592±0.05	0.634±0.03	0.691±0.06	0.685±0.04	**0.707±0.04**
	DT	0.614±0.06	0.54±0.02	0.635±0.05	0.593±0.07	**0.668±0.05**	0.61±0.06	0.628±0.01
	KNN	0.675±0.04	0.734±0.03	0.662±0.05	0.676±0.04	0.797±0.04	**0.816±0.03**	0.773±0.02
TOX_171	SVM	0.658±0.03	0.731±0.01	0.839±0.01	0.783±0.02	0.955±0.01	0.988±0.01	**0.993±0.01**
	NB	0.609±0.03	0.619±0.01	0.687±0.03	0.707±0.02	0.741±0.02	0.752±0.02	**0.788±0.02**
	DT	0.551±0.04	0.555±0.03	0.594±0.03	0.557±0.03	0.6±0.02	0.599±0.03	**0.616±0.02**
	KNN	0.739±0.02	0.785±0.03	0.886±0.03	0.809±0.03	0.946±0.01	0.964±0.01	**0.973±0.01**
leukemia	SVM	0.959±0.01	0.96±0.01	0.954±0.01	0.961±0.01	0.967±0.01	0.97±0.01	**0.976±0.01**
	NB	0.947±0.01	0.934±0.01	0.93±0.01	0.934±0.01	0.94±0.01	0.943±0.02	**0.947±0.01**
	DT	0.931±0.01	0.935±0.01	0.943±0.01	0.948±0.01	0.938±0.01	0.932±0.02	**0.94±0.01**
	KNN	0.965±0.01	0.951±0.01	0.95±0.01	0.956±0.01	0.966±0.01	0.964±0.01	**0.97±0.01**
ALLAML	SVM	0.943±0.01	0.964±0.01	0.969±0.01	0.968±0.01	0.94±0.01	**0.97±0.01**	0.967±0.01
	NB	0.958±0.01	0.957±0.01	0.947±0.02	0.956±0.01	0.955±0.02	0.966±0.01	**0.968±0.01**
	DT	0.86±0.04	0.855±0.02	0.867±0.04	0.854±0.03	0.851±0.03	0.862±0.03	**0.867±0.03**
	KNN	0.901±0.02	0.946±0.01	0.922±0.01	0.926±0.01	0.933±0.01	0.944±0.02	**0.96±0.01**
pixraw10P	SVM	0.422±0.06	0.484±0.06	0.761±0.06	0.756±0.04	0.945±0.01	0.95±0.01	**0.951±0.02**
	NB	**0.869±0.04**	0.607±0.04	0.668±0.03	0.663±0.04	0.828±0.01	0.852±0.04	0.727±0.04
	DT	**0.543±0.06**	0.482±0.07 ..	0.54±0.08	0.493±0.05	0.511±0.06	0.419±0.06	0.468±0.04
	KNN	0.867±0.02	0.939±0.01	0.834±0.04	0.858±0.04	0.923±0.02	0.935±0.02	**0.958±0.01**
arcene	SVM	0.593±0.02	0.708±0.01	0.796±0.02	0.799±0.02	0.811±0.02	0.825±0.01	**0.836±0.01**
	NB	0.575±0.01	0.678±0.01	0.635±0.03	0.678±0.03	0.746±0.02	0.732±0.01	**0.753±0.02**
	DT	0.589±0.02	0.672±0.02	0.728±0.03	**0.754±0.02**	0.694±0.03	0.728±0.02	0.699±0.04
	KNN	0.573±0.03	0.706±0.02	0.808±0.02	0.81±0.01	0.818±0.02	0.84±0.02	**0.842±0.01**
W/T/L		5/0	0/0	0/0	2/0	3/0	2/0	27/0

**Table 6:** *F*_1_-score of 10-fold cross validation on 10 datasets (Mean±std). Selected 50 features.

Dataset		Chi-square	Fisher-score	ReliefF	RBEFF	NCFS	RB-NCFS	KNCFS
SCADI	SVM	0.665±0.01	0.799±0.01	0.795±0.01	**0.809±0.01**	0.8±0.01	0.802±0.02	0.803±0.01
	NB	0.635±0.02	0.775±0.02	0.76±0.01	0.774±0.02	0.754±0.02	0.773±0.01	**0.778±0.02**
	DT	0.698±0.01	0.769±0.04	0.764±0.02	**0.781±0.03**	0.757±0.04	0.768±0.02	0.759±0.03
	KNN	0.697±0.02	0.789±0.01	0.79±0.02	**0.8±0.01**	0.79±0.02	0.788±0.02	0.794±0.02
lung_discrete	SVM	0.786±0.03	0.755±0.01	0.783±0.03	0.787±0.02	0.8±0.03	0.797±0.03	**0.802±0.03**
	NB	0.515±0.03	0.484±0.05	0.556±0.03	0.541±0.04	0.526±0.03	0.519±0.03	**0.581±0.04**
	DT	0.538±0.05	0.538±0.03	0.519±0.05	0.514±0.04	0.591±0.06	0.557±0.05	**0.589±0.07**
	KNN	0.831±0.03	0.81±0.03	0.845±0.04	0.852±0.03	**0.858±0.02**	0.836±0.04	**0.858±0.03**
warpAR10P	SVM	0.596±0.03	0.755±0.02	0.677±0.03	0.678±0.02	0.943±0.02	0.951±0.01	**0.965±0.01**
	NB	0.707±0.02	0.713±0.02	0.644±0.02	0.642±0.01	0.818±0.02	0.815±0.03	**0.822±0.04**
	DT	**0.68±0.03**	0.632±0.03	0.628±0.04	0.631±0.02	0.438±0.02	0.566±0.04	0.526±0.06
	KNN	0.721±0.03	0.793±0.03	0.766±0.02	0.763±0.02	0.874±0.04	0.903±0.02	**0.94±0.02**
tumors_C	SVM	**0.66±0.04**	0.658±0.03	0.633±0.02	0.595±0.03	0.522±0.04	0.501±0.04	0.638±0.06
	NB	0.656±0.03	0.616±0.02	0.619±0.05	0.598±0.03	0.553±0.06	0.508±0.08	**0.664±0.06**
	DT	**0.609±0.07**	0.576±0.06	0.577±0.05	0.594±0.05	0.495±0.05	0.521±0.04	0.571±0.09
	KNN	0.574±0.04	**0.646±0.05**	0.578±0.04	0.526±0.04	0.561±0.05	0.502±0.04	0.574±0.05
GLIOMA	SVM	0.706±0.04	0.607±0.06	0.662±0.02	0.674±0.03	0.769±0.03	0.772±0.03	**0.779±0.02**
	NB	0.499±0.05	0.621±0.04	0.608±0.04	0.619±0.04	0.658±0.04	0.678±0.04	**0.68±0.05**
	DT	0.595±0.07	0.63±0.06	0.604±0.06	0.587±0.09	0.621±0.05	0.619±0.08	**0.663±0.06**
	KNN	0.73±0.04	0.684±0.03	0.687±0.03	0.688±0.05	0.794±0.03	0.793±0.03	**0.804±0.02**
TOX_171	SVM	0.741±0.03	0.585±0.05	0.86±0.02	0.831±0.02	0.992±0.01	0.991±0.01	**0.996±0.01**
	NB	0.628±0.02	0.6±0.04	0.712±0.02	0.742±0.02	0.804±0.04	0.8±0.02	**0.827±0.03**
	DT	0.565±0.04	0.507±0.07	0.557±0.05	0.556±0.03	0.606±0.04	**0.649±0.05**	0.62±0.03
	KNN	0.834±0.02	0.658±0.06	0.904±0.03	0.873±0.02	**0.983±0.02**	0.953±0.02	**0.983±0.01**
leukemia	SVM	0.973±0.01	0.976±0.01	0.957±0.01	0.964±0.01	0.973±0.01	0.978±0.01	**0.98±0.01**
	NB	**0.95±0.01**	0.929±0.01	0.925±0.01	0.928±0.02	0.929±0.01	0.932±0.02	0.931±0.02
	DT	0.939±0.02	0.937±0.01	0.94±0.01	0.927±0.02	0.939±0.01	0.94±0.02	**0.946±0.01**
	KNN	0.966±0.01	0.963±0.01	0.955±0.01	0.953±0.01	0.974±0.01	**0.981±0.01**	0.98±0.01
ALLAML	SVM	0.966±0.01	0.959±0.01	0.96±0.01	0.962±0.01	0.728±0.03	0.966±0.01	**0.969±0.01**
	NB	0.955±0.01	0.958±0.01	0.959±0.01	0.956±0.02	0.773±0.02	0.964±0.01	**0.98±0.01**
	DT	0.852±0.03	0.862±0.03	**0.863±0.02**	0.852±0.03	0.722±0.04	0.838±0.03	0.841±0.03
	KNN	0.914±0.01	0.937±0.02	0.924±0.02	0.924±0.02	0.741±0.03	0.937±0.01	**0.954±0.01**
pixraw10P	SVM	0.532±0.03	0.539±0.04	0.824±0.04	0.791±0.06	0.962±0.01	0.95±0.01	**0.959±0.01**
	NB	0.803±0.03	0.429±0.04	0.554±0.03	0.543±0.04	**0.805±0.04**	0.797±0.05	0.804±0.05
	DT	**0.492±0.06**	0.462±0.03	0.464±0.04	0.468±0.03	0.414±0.08	0.314±0.04	0.428±0.06
	KNN	0.906±0.01	0.941±0.01	0.909±0.04	0.894±0.05	0.939±0.01	0.949±0.01	**0.951±0.01**
arcene	SVM	0.65±0.01	0.709±0.01	0.79±0.01	0.819±0.02	0.815±0.02	**0.843±0.02**	0.835±0.02
	NB	0.624±0.01	0.675±0.01	0.703±0.02	0.719±0.02	0.768±0.01	0.758±0.01	**0.78±0.02**
	DT	0.637±0.02	0.699±0.03	0.725±0.03	0.739±0.02	0.719±0.02	**0.74±0.03**	0.708±0.04
	KNN	0.648±0.02	0.716±0.02	0.808±0.02	0.837±0.02	0.838±0.01	**0.859±0.02**	0.844±0.02
W/T/L		4/0	1/0	1/0	3/0	1/2	7/0	22/2

Regarding classification accuracy,KNCFS achieved the best results 27 times for selecting 50 features and 26 times(21/5) for selecting 100 features, Fig 3 shows that in most cases,KNCFS outperforms the other six comparative methods in terms of classification accuracy,and Fig 4 (sub-figure a) also shows that KNCFS obtains the best classification accuracy.It’s worth noting that RB-NCFS (2/0 and 8/1) generally performs better than NCFS (0/0 and 1/1) because it is a random subspace method that considers sample diversity,giving it an advantage over traditional methods.However, due to its lack of a solution for feature collinearity,its performance falls short compared to KNCFS.

On the other hand,KNCFS obtained the best results 27 times in Table 5 and 25 times(22/2) in Table 6 for *F*_1_-score,demonstrating a significant advantage in *F*_1_-score as well, and, Fig 4 (sub-figure b) also shows that KNCFS obtains the best *F*_1_-score. RBEFF achieved the best results twice (selecting 50 features) and three times (selecting 100 features) in the SCADI dataset because of its lower dimensionality. RBEFF is a filter-based feature selection method that performs well on small datasets. However,due to its lack of guidance algorithms in the feature selection stage and its inability to consider nonlinear relationships between features,its performance on high-dimensional datasets falls short compared to KNCFS.

#### 4.5.3 Success rate of feature selection

**Fig 5:**
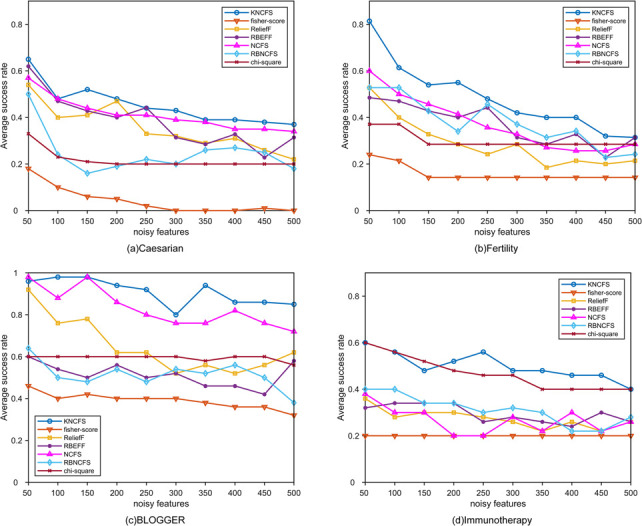
Success rate of feature selection on synthetic datasets.

In this subsection,we generated synthetic datasets based on four real-wrold datasets from the UCI database.These datasets are as follows:Caesarian(80 samples, 5 features,2 classes),Fertility(100 samples,10 features,2 classes),BLOGGER(100 samples,5 features,2 classes) and Immunotherapy(90 samples,7 features,2 classes). Before starting the experiment,we considered the initial features of each dataset as relevant.We then added noise features consisting of random numbers with a mean of 0 and a variance of 5.The number of noise features varies from 50 to 500 in increments of 50 to form a set of synthetic datasets.We apply all seven methods to each synthetic dataset to learn the importance of the features and then rank the importance of the features.For example,on the Caesarian dataset with five relevant features,we ranked the feature weights and counted the number of relevant features in the top five to measure the success of feature selection.The experimental results for the synthetic dataset are shown in Fig 5.

In the Caesarean dataset (sub-figure a),KNCFS and Chi-square have similar results when the number of noisy features is below 200.On the Fertility dataset (sub-figure b),KNCFS outperforms NCFS slightly,and the gap between KNCFS and RBEFF widens as the number of noisy features increases.Lastly,KNCFS consistently exhibits the strongest performance on the BLOGGER and Immunotherapy datasets. This study establishes KNCFS as a compelling contender.RB-NCFS is often less effective than NCFS,possibly due to overfitting caused by random noise features interfering with the random subspace approach.This creates bias in the capability of RB-NCFS to resolve significant features,resulting in a lower FS success rate than that of NCFS and KNCFS.KNCFS solves the collinearity issue in random multi-subspace learning methods,enabling it to achieve the best results in terms of feature selection success rate.

### 4.6 Parameter sensitivity analysis

**Fig 6:**
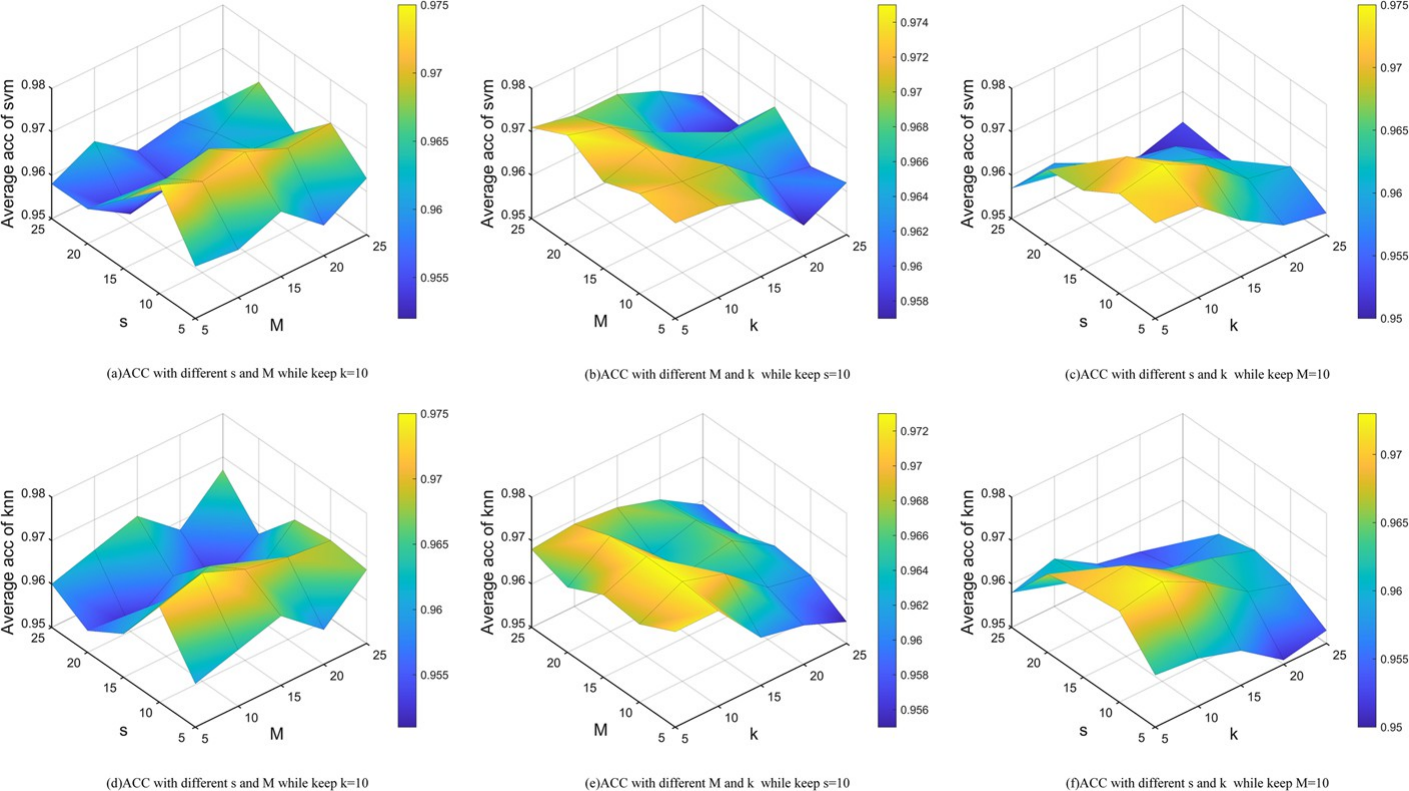
The acc with the parameters *s*,*M*,*k* in leukaemia dataset on KNN and svm classification.

**Fig 7:**
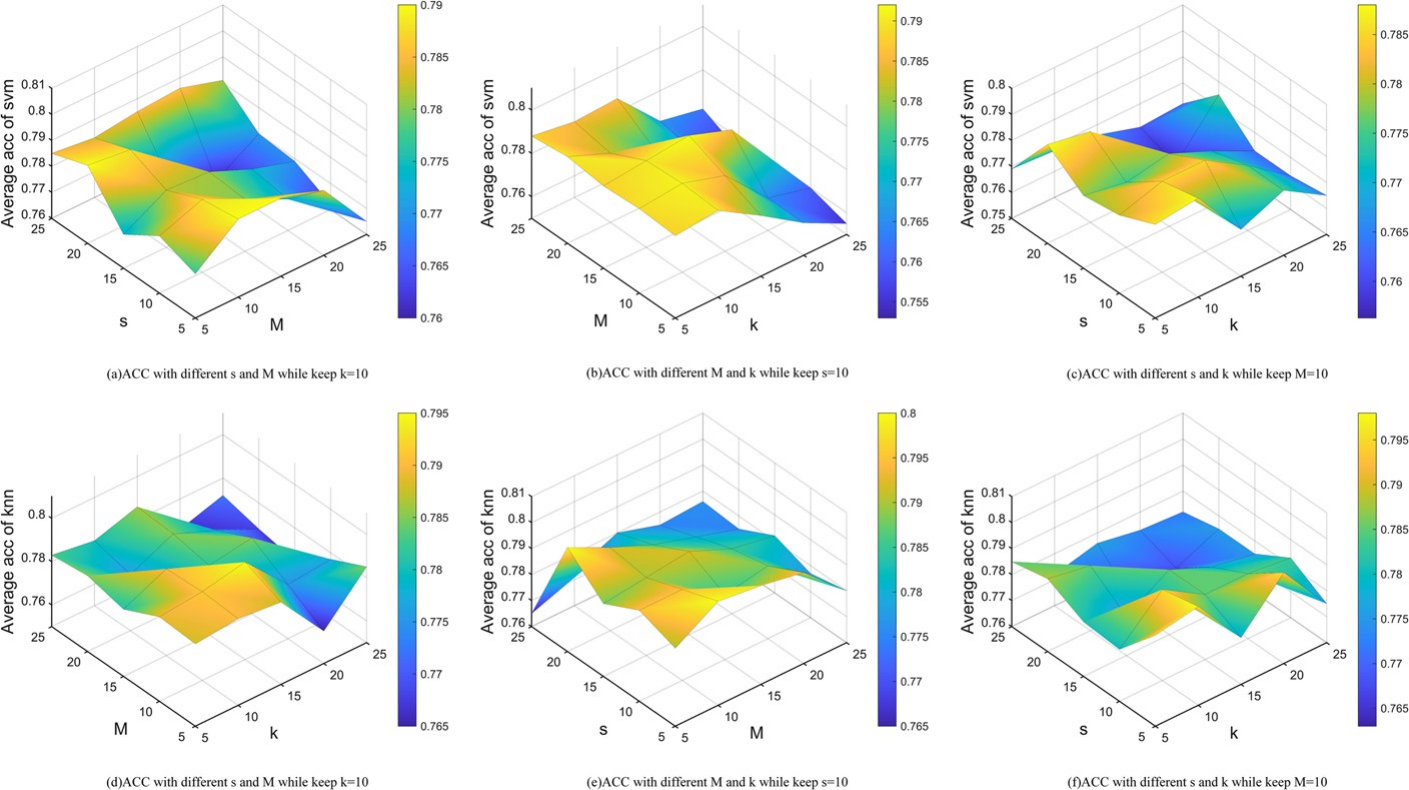
The acc with the parameters *s*,*M*,*k* in GLIOMA dataset on KNN and svm classification.

In order to investigate the influence of the parameters *M*,*s* and *k* on the performance of our proposed method,we performed sensitivity experiments on the average accuracy of the SVM and KNN classifiers.For simplicity,we selected two representative datasets,namely "leukaemia" and "GLIOMA",to participate in the parameter sensitivity analysis.As shown in Figs 6 and 7,the accuracy of the algorithm decreases when *k* exceeds 15,with the highest accuracy achieved when *k* is in the range {5, 10}.Similarly,the algorithm shows higher accuracy when s is in the range {5, 10}.Overall,variations in *M* have a relatively small effect on the accuracy of the algorithm.Therefore,it can be concluded that the algorithm is not very sensitive to the value of *M*.Finally,we set *M*,*s* and *k* to {10, 10, 10}.

## 5. Conclusion

Feature selection (FS) is an important data preprocessing technique that reduces the dimensionality of a dataset,decreases model complexity and lowers computational cost.In this paper,we propose a random multi-subspace method based on feature correlation clustering,which is implemented through an iterative process consisting of two key phases:a stochastic subspace learning phase and a feature vector weighting phase.The stochastic subspace learning phase aims to increase the diversity of samples to extract more information, while the feature vector weighting phase evaluates the feature partitions.We conducted numerical experiments on two types of datasets:real-world datasets and synthetic datasets with noisy features.The experimental results,when compared with Chi-square,Fisher-score,ReliefF,RBEFF, NCFS and RB-NCFS,show that KNCFS is a state-of-the-art FS algorithm as it can effectively identify relevant features.In this study,we used the existing feature selection method NCFS in the subspace learning phase,but there are more advanced feature selection methods that can be used to improve the effectiveness of the algorithm.In addition,selecting feature selection methods or unsupervised feature selection algorithms capable of handling multi-labeled data could further extend the applicability of the algorithm.Feature selection remains an important area of research with many other aspects to be explored.
